# Supplementary material for: Bitter Orange (Citrus aurantium Linné) Improves Obesity by Regulating Adipogenesis and Thermogenesis through AMPK Activation
Source: Nutrients. 2019 Aug 22;11(9):1988. doi: 10.3390/nu11091988 (PMC6770725; doi:10.3390/nu11091988)
Supplement: Supplementary file 1 [file nutrients-11-01988-s001.pdf]

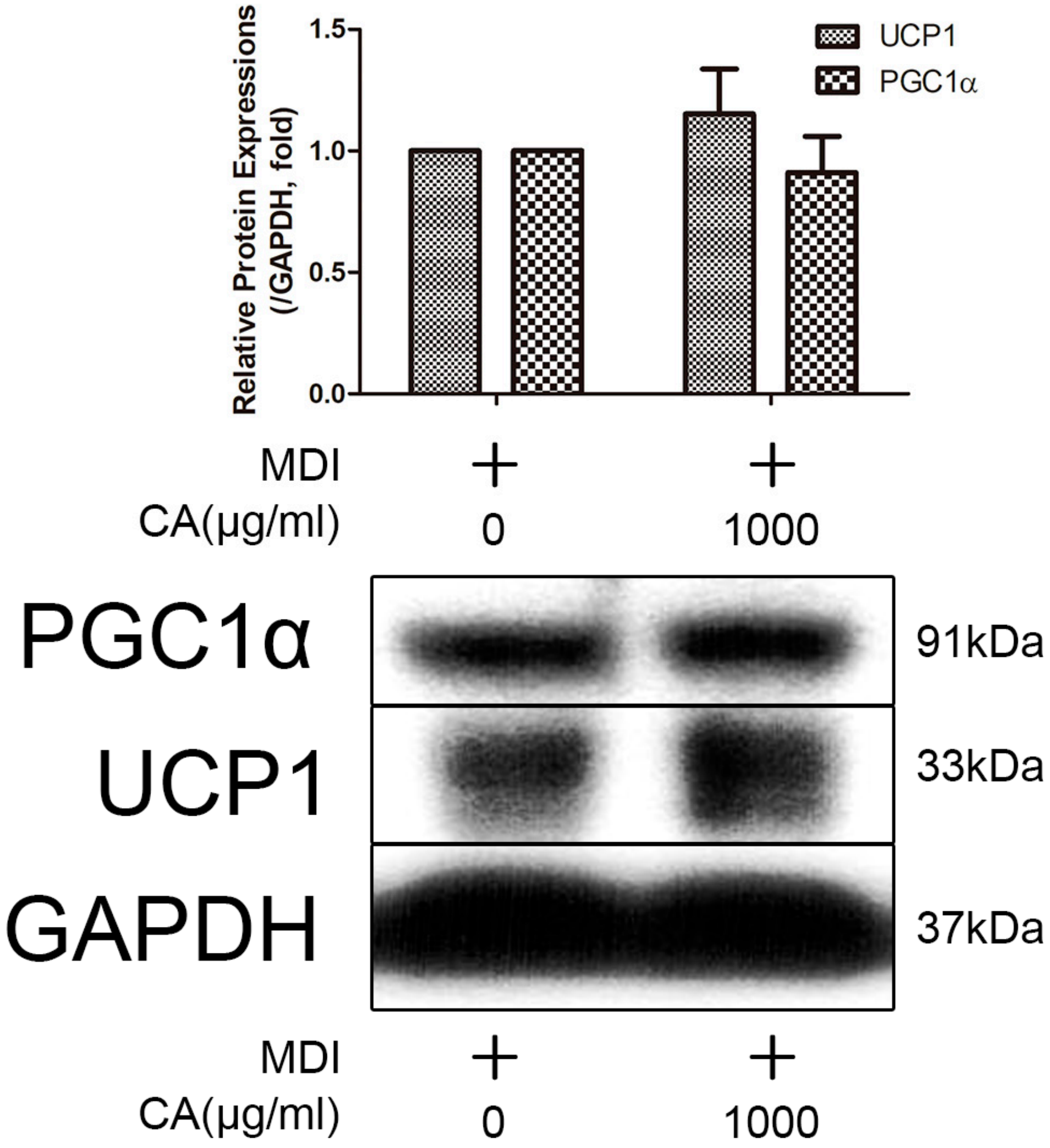

Supplementary Figure S1. Effect of CA on browning of 3T3-L1 adipocytes. Western blot analysis of UCP1 and PGC1*α* were performed in 3T3-L1 treated with or without CA. Results were expressed relative to GAPDH. MDI, differentiation medium; CA, bitter orange (*Citrus aurantium* Linné).

Supplementary Table S1. Composition of experimental diets (g/kg).

**Formula**	**NC**	**HFD**
Casein	200.0	265.0
L-Cystine	3.0	4.0
Corn Starch	397.486	-
Maltodextrin	132.0	160.0
Sucrose	100.0	90.0
Lard	-	310.0
Soybean Oil	70.0	30.0
Cellulose	50.0	65.5
Mineral Mix[a]	35.0	48.0
Calcium Phosphate	-	3.4
Vitamin Mix[b]	10.0	21.0
Choline Bitartrate	2.5	3.0
TBHQ, antioxidant[c]	0.014	-
Blue Food Color	-	0.1

[a]Mineral Mix, AIN-93G-MX (94046) containing (g/kg): calcium phosphate dibasic 500, sodium chloride 74, potassium citrate 220, potassium sulfate 52, magnesium oxide 24, manganous carbonate 3.5, ferric citrate 6, zinc carbonate 1.6, cupric carbonate 0.3, potassium iodate 0.0.1, sodium selenite 0.01, chromium potassium sulfate 0.55

[b]Vitamin Mix, AIN-93-VX (94047) containing (g/kg): thiamin HCl 0.6, riboflavin 0.6, pyridoxine HCl 0.7, niacin 3, calcium pantothenate 1.6, folic acid 0.2, biotin 0.02, vitamin B12 (0.1 % trituration in mannitol) 1, dry vitamin A palmitate (500,000 U/g) 0.25, manadione sodium bisulfite complex 0.15

[c]TBHQ: tertiary butylhydroquinone

Supplementary Table S2. The primer sequences used for Real-Time RT-PCR.

Gene	Primer sequences	Accession No. (GenBank)
Pparg	5′-TGCCAGTACTGCCGTTTTCA-3′ (sense)<br>5′-GCGAATTGCATTGTGTGACAT-3′ (antisense)	NM_001127330
Cebpa	5′-GCCGAGATAAAGCCAAACAA-3′ (sense)<br>5′-CCTTGACCAAGGAGCTCTCA-3′ (antisense)	NM_001287514
Fabp4	5′-CGTAAATGGGGATTTGGTCA-3′ (sense)<br>5′-TCGACTTTCCATCCCACTTC-3′ (antisense)	NM_024406
Adipoq	5′-AGACCTGGCCACTTTCTCCTCATT-3′ (sense)<br>5′-AGAGGAACAGGAGAGCTTGCAACA-3′ (antisense)	NM_009605
Retn	5′-TTCCTTGTCCCTGAACTGCT-3′ (sense)<br>5′-AGCTCAAGACTGCTGTGCCT-3′ (antisense)	NM_001204959
Pgc1a	5′-AATGCAGCGGTCTTAGCACT-3′ (sense)<br>5′-TGTTGACAAATGCTCTTCGC-3′ (antisense)	NM_008904
Ucp1	5′-AACTGTACAGCGGTCTGCCT-3′ (sense)<br>5′-TAAGCCGGCTGAGATCTTGT-3′ (antisense)	NM_009463
Prdm16	5′-TGGGCTCACTACCCTACCAC-3′ (sense)<br>5′-GAC TTT GGC TCA GCC TTG AC-3′ (antisense)	NM_001291026
Sirt3	5′-TCGAAGGAAAGATGTGGTCC-3′ (sense)<br>5′- ATCTGTCCTGTCCATCCAGC-3′ (antisense)	NM_002433
Cidea	5′-TCAGACCTTAAGGGACAACACGCA-3′ (sense)<br>5′-TTCTTTGGTTGCTTGCAGACTGGG-3′ (antisense)	NM_007702
Cytc	5′-TTGTTCAGAAGTGTGCCCAG-3′ (sense)<br>5′-CCAGGTGATGCCTTTGTTCT-3′ (antisense)	NM_007808
Gapdh	5′-AACTTTGGCATTGTGGAAGG-3′ (sense)<br>5′-GGATGCAGGGATGATGTTCT-3′ (antisense)	NM_001289726
